# Modeling Frequency Reduction in Human Groups Performing a Joint Oscillatory Task

**DOI:** 10.3389/fpsyg.2021.753758

**Published:** 2022-01-04

**Authors:** Carmela Calabrese, Benoît G. Bardy, Pietro De Lellis, Mario di Bernardo

**Affiliations:** ^1^Department of Electrical Engineering and Information Technology, University of Naples Federico II, Naples, Italy; ^2^EuroMov Digital Health in Motion, University of Montpellier IMT Mines Ales, Montpellier, France

**Keywords:** joint action, human behavior, modeling, Kuramoto oscillators, slowing down

## Abstract

In human groups performing oscillatory tasks, it has been observed that the frequency of participants' oscillations reduces when compared to that acquired in solo. This experimental observation is not captured by the standard Kuramoto oscillators, often employed to model human synchronization. In this work, we aim at capturing this observed phenomenon by proposing three alternative modifications of the standard Kuramoto model that are based on three different biologically-relevant hypotheses underlying group synchronization. The three models are tuned, validated and compared against experiments on a group synchronization task, which is a multi-agent extension of the so-called mirror game.

## 1. Introduction

Joint action can be regarded as any form of embodied social interaction where two or more individuals tend to coordinate their movements, often in a highly synchronized way in space and time, in order to reach a common goal. This phenomenon is observed in several everyday scenarios, including music ensembles (Loehr et al., 2011) and team rowing (Cuijpers et al., 2019), and has potential implications in group rehabilitation (Virta et al., 2008; Calabrese et al., 2021).

Physics and social science offer several mathematical frameworks to describe collective oscillatory perceptuo-motor behaviors in joint action (Néda et al., 2000; Sumpter, 2006; Castellano et al., 2009; Ashwin et al., 2016). Namely, both the Haken-Kelso-Bunz (HKB) and the heterogeneous Kuramoto oscillators have been successfully employed to explain how synchronization emerges in human groups performing oscillatory tasks (Kuramoto, 1984; Haken et al., 1985). For instance, Alderisio et al. (2017a) found that the Kuramoto model was able to capture the type and level of group coordination that was experimentally observed, depending on group homogeneity and the visual coupling among group members. Although effective in reproducing the level of synchronization in the group, this model predicts synchronization of the group oscillatory motion to the average value of the individual characteristic frequencies when playing solo. This is in contrast with the joint-action literature that shows how cooperative actions require a more selective and slower mechanism compared to individual movements (Cavallo et al., 2014).

Slowing down of individuals' motion when coordinating with others has been widely observed across several tasks, including applause (Néda et al., 2000), where it has been observed that synchronization is achieved through a period doubling of the clapping rhythm, or in finger tapping, where participants were found to tap faster alone than when involved in rhythmic cooperative tasks (Coey et al., 2016), as well as in human-robot interactions (Lorenz et al., 2011). In addition, a recent experimental study on intentional group synchronization showed that humans reduce the frequency of oscillation of their fingers when they are asked to attain unison in space and time (Bardy et al., 2020), thereby suggesting that individuals modulated their behavior to maximize perceptual coupling and increase their level of synchronization.

Motivated by these experimental findings, we propose three new versions of the standard Kuramoto model, each of them anchored into a specific and functionally relevant hypothesis. The goal is to obtain a model that is able to capture at the same time (i) the level of coordination observed in human groups performing oscillatory tasks, and (ii) the reduction in the frequency of oscillation when compared to a solo performance. After developing three alternative models built around different biologically-relevant explanations of the slowing down phenomenon, we test them against experiments on a group synchronization task, a multi-agent extension of the so-called mirror game (Noy et al., 2011). Specifically, using the three collected datasets, we experimentally tuned, validated, and compared the three models, in terms of their ability to match the synchronization levels and oscillation frequency reduction observed in the experiments.

## 2. Synchronization Metrics

In what follows, we quantify the level of coordination in a group of *N* players, whose phases at time *t* are denoted as θ_1_(*t*), …, θ_*N*_(*t*). The *order parameter* 0 ≤ *r*(*t*) ≤ 1 describing the phase cohesiveness of the group at time *t* is defined as follows:


(1) $$\begin{array}{l} r\left(t\right)=|q\left(t\right)|, \end{array}$$

where


(2) $$\begin{array}{l} q\left(t\right)=\frac{1}{N}\overset{N}{\underset{i=1}{\sum }}e^{j\theta_{i}\left(t\right)}, \end{array}$$

whereas the phase


(3) $$\begin{array}{l} \psi \left(t\right)={\text{tan}}^{-1}\frac{\text{Im}\left\{q\left(t\right)\right\}}{\text{Re}\left\{q\left(t\right)\right\}} \end{array}$$

associated to the order parameter will be the *group phase* at time *t*. The order parameter quantifies the level of phase synchronization in the group, with *r*(*t*) = 1 corresponding to the players sharing the same phase at time *t*.

The *level of frequency coordination* of the entire ensemble at time *t* can be quantified as


(4) $$\begin{array}{l} \rho \left(t\right):=\frac{1}{N}|\overset{N}{\underset{i=1}{\sum }}e^{j\Delta \phi_{i}\left(t\right)}|, \end{array}$$

where $\Delta \phi_{i}\left(t\right):=\phi_{i}\left(t\right)-{\bar{\phi }}_{i}\left(t\right)$, with


(5) $$\begin{array}{l} {\bar{\phi }}_{i}\left(t\right):=\tan^{-1}\frac{\text{Im}\left\{{\bar{\phi }}_{i}^{\prime }\right\}}{\text{Re}\left\{{\bar{\phi }}_{i}^{\prime }\right\}}. \end{array}$$

Differently from Richardson et al. (2012), ${\bar{\phi }}_{i}^{\prime }$ is the moving average over a time window *w* of the relative phase ϕ_*i*_(*t*): = θ_*i*_(*t*)−ψ(*t*) of oscillator *i* with respect to the group, that is,


(6) $$\begin{array}{l} {\bar{\phi }}_{i}^{\prime }\left(t\right):=\frac{1}{w}\overset{t}{\underset{l=t-w}{\sum }}e^{j\phi_{i}\left(l\right)}, \end{array}$$

The index 0 ≤ ρ(*t*) ≤ 1 gives information on the variability of the phase mismatch among all oscillators. Namely, ρ(*t*) equal to 1 corresponds to a perfect matching of the oscillation frequencies at time *t*.

## 3. Modeling Human Group Synchronization

The emergence of synchronization in interacting groups of humans performing oscillatory tasks has been successfully captured by networks of nonlinearly coupled heterogeneous Kuramoto oscillators (Kuramoto, 1984; Alderisio et al., 2017a):


(7) $$\begin{array}{l} {\overset{∙}{\theta }}_{i}\left(t\right)=\omega_{i}+c\overset{N}{\underset{j=1}{\sum }}\sin\left(\theta_{j}\left(t\right)-\theta_{i}\left(t\right)\right), \end{array}$$

where θ_*i*_ is the phase associated to the motion of player *i*, ω_*i*_ represents its natural frequency, and *c* the coupling gain describing the intensity of the interaction between the agents. However, this standard model predicts frequency synchronization onto the average value of the individual characteristic frequencies (Bullo, 2018), at a distance from the experimental observation of a frequency reduction in group oscillations (Lorenz et al., 2011; Cavallo et al., 2014; Coey et al., 2016; Bardy et al., 2020), thus suggesting that model (Equation 7) needs to be appropriately modified.

Toward capturing this frequency reduction, we propose three different extensions of the standard Kuramoto model, each acting on one of the three salient components of a complex system, that is, the *individual dynamics*, the *interaction topology*, and the *communication protocol*. Each of the extensions, which we will call Model 1, 2, and 3, respectively, is based on one of three main biologically-relevant explanations of the observed frequency reduction (Foulkes and Miall, 2000; Chafe et al., 2010; Serences and Kastner, 2014). In particular, the first two models will relate this phenomenon to behavioral plasticity, that is, the ability each individual has of adjusting to complex environmental conditions, whereas the latter to the inherent perception-action delays.

### 3.1. Model 1: Behavioral Plasticity as the Result of Individual Adaptability

Behavioral plasticity is crucial to achieve successful coordination in humans, and it has been posited that such human ability is the result of movement adaptation (Van Der Steen and Keller, 2013), whereby our motor system needs to deal with muscular fatigue, external loads, or changes in our sensory systems guiding the movement (Foulkes and Miall, 2000). This adaptation is often associated with a slower individual motion, which favors synchronization and fosters a successful interaction with others (Van Braeckel et al., 2007). From a social perspective, interpersonal entrainment leads to de-individuation and to the formation of a common group identity amongst partners, motivating the individuals to adapt their behavior (Cross et al., 2019).

Here, we model the observed frequency reduction as the result of an adaptive mechanism where individuals in the group reduce their natural frequencies until a desired degree of phase synchronization is achieved. This is quantified by a threshold value $\tilde{r}>0$ of the order parameter (Equation 1), so that model (Equation 7) becomes


(8) $$\begin{array}{l} \begin{array}{ll} {\overset{∙}{\theta }}_{i}\left(t\right) & =\omega_{i}\left(t\right)+c\overset{N}{\underset{j=1}{\sum }}\sin\left(\theta_{j}\left(t\right)-\theta_{i}\left(t\right)\right),\\ {\overset{∙}{\omega }}_{i}\left(t\right) & =\{\begin{array}{ll} -\frac{1}{r^{2}\left(t\right)}\omega_{i}\left(t\right), & \text{if }r\left(t\right)<\tilde{r},\\ 0, & \text{otherwise}. \end{array} \end{array} \end{array}$$

Note that the farther the group is from the desired level of coordination $\tilde{r}$, the larger the decrease in individual frequencies will be.

### 3.2. Model 2: Behavioral Plasticity as a Result of Selective Attention

An alternative explanation of human behavioral plasticity lies in *selective attention*, which is the ability to focus on one source of information while disregarding the others (Portas et al., 1998). This neural mechanism allows to complete group tasks successfully (Capozzi et al., 2016), coping with i) the noise affecting the sensory neurons that encode external stimuli, and ii) the fact that only the most relevant visual stimuli can be processed and translated into actions by the motor system (Serences and Kastner, 2014).

Both factors reduce the speed and accuracy of perception-action responses, and thus we propose to weigh the interactions among the individuals to prioritize the relevant stimuli:


(9) $$\begin{array}{l} {\overset{∙}{\theta }}_{i}\left(t\right)=\omega_{i}+c\overset{N}{\underset{j=1}{\sum }}w_{ij}\sin\left(\theta_{j}\left(t\right)-\theta_{i}\left(t\right)\right). \end{array}$$

In this expression, *w*_*ij*_≥0 quantifies the attention level that agent *i* devotes to the motion of agent *j*. Here, we ground the selection of these weights in the theory of *motor variability* (Bernstein, 1966), which is viewed as the result of adaptive and compensatory mechanisms to e.g., cope with perturbations, reduce injury risks, or improve coordination (Bardy and Laurent, 1998; Bartlett et al., 2007). In oscillatory tasks, motor variability in each individual, say *i*, can be simply quantified in terms of the standard deviation σ_*i*_ of the oscillatory frequency (Longo and Meulenbroek, 2018), which we computed from experiments performed by a single individual (from now on denoted as the *solo* experimental condition). We then hypothesize that agents with a larger standard deviation will be more prone to adjust their rhythm to that of their neighbors. At the same time, recent findings indicated that individuals involved in a joint action adjust the variability of their own movements depending on the predictability of their partners' movements (Sabu et al., 2020), thus suggesting that the attention that agent *i* devotes to agent *j* is inversely proportional to the standard deviation σ_*j*_. Accordingly, we propose to select *w*_*ij*_ in Equation (9) as


(10) $$\begin{array}{l} w_{ij}=\frac{\sigma_{i}}{\sigma_{j}}. \end{array}$$

Indeed, our hypothesis here is that the standard deviation in solo is the proxy of the individual motor signature (Slowinski et al., 2016) of each individual, thereby the ratio between these standard deviation is what the individual actually perceives when interacting with the others.

### 3.3. Model 3: Perception-Action Delays

Previous work has considered the presence of delays in the neuro-communication pathways for modeling oscillatory behavior (Izhikevich, 2007; Timms and English, 2014; Petkoski and Jirsa, 2019; Slowinski et al., 2020). In fact, multilevel crosstalk represents an important neural basis for motor control (Banerjee and Jirsa, 2007), since multi-sensory processing is not instantaneous and involves participation of different senses (e.g., vision, hearing) to facilitate the perception of environmental stimuli (Thakur et al., 2016).

As a third alternative, we propose to explain the reduced frequency observed in groups of individuals synchronizing their movements by introducing a perception-action delay in the standard Kuramoto model (Equation 7) as follows:


(11) $$\begin{array}{l} {\overset{∙}{\theta }}_{i}\left(t\right)=\omega_{i}+c\overset{N}{\underset{j=1}{\sum }}\sin\left(\theta_{j}\left(t-\tau \right)-\theta_{i}\left(t\right)\right), \end{array}$$

where the neuro-motor delays are captured by the parameter τ, which corresponds to the time required by an agent *i* to track the position of an agent *j*≠*i*, process this information, and modulate its own action accordingly. Estimating the delay associated with perception-action has been the subject of extensive literature in neuroscience (Clarke et al., 1999), psychology (Brown et al., 1999), and behavioral science (Marzi et al., 1991; Li et al., 2012), with all studies agreeing that τ should lie in the range [5, 300] × 10^−3^ s. Note that the delay might be also modeled through a phase shift in the coupling function (Izhikevich, 2007), but we preferred to explicitly model it to clarify that it is a perception delay, whereby in Equation (11) the delay parameter τ affects θ_*j*_ (the phase of individual *j* perceived with delay τ by individual *i*) and not θ_*i*_ (the own phase of individual *i*).

## 4. Methods

### 4.1. Data Collection

**Figure 1:**
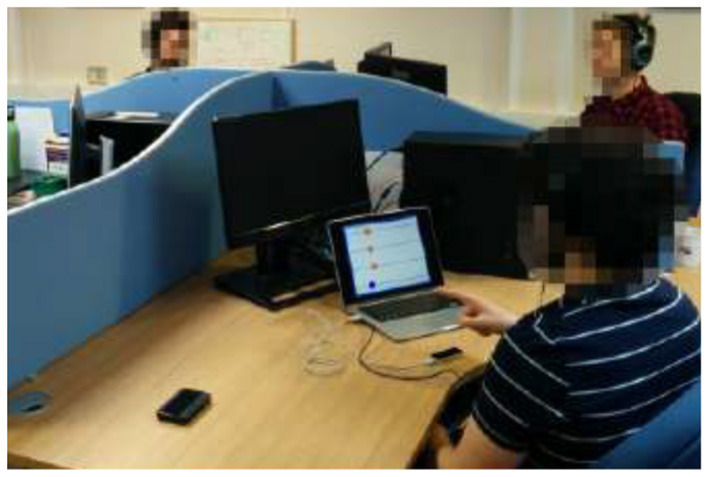
Experimental setup- Chronos. Participants had to oscillate and synchronize the index finger of their preferred hand over a Leap Motion controller while being virtually connected with the others through the platform Chronos.

We considered a dataset coming from experiments on group motor coordination performed at the University of Naples Federico II. The experiments were run via the computer-based architecture *Chronos* (Alderisio et al., 2017b), which allows remote motor coordination between players in the absence of social (visual and/or acoustic) interaction. The Chronos platform is a computer-based architecture consisting of different software/hardware devices. A central server unit receives position data from the clients (i.e., players), captured by a low-cost position sensor- Leap Motion device (Guna et al., 2014). The positions of each agent are then broadcast to the others, through a Wi-Fi network, and appear on the monitor of each individual personal computer (see Figure 1).

*Task description*. The dataset referred to experiments with groups of 5 participants that were asked to move their index finger on a Leap Motion controller so as to move a ball on the screen representing their own avatar, oscillating from left to right and vice versa. Each experiment started with a 30 s solo session to capture the natural frequency of each individual, identified as its average frequency across 7 solo trials, see Table 1. The second session of the experiments (6 trials, 30 s each) was devoted to investigating group synchronization. Participants were connected through the software platform Chronos and were asked to oscillate their fingers in synchrony with the others. Namely, they were instructed to “Synchronize the movement of your finger from left to right with the movement of the others, as naturally as possible, as if you could do it for 30 min” (see Figure 1). A demonstration was performed to make sure the task was understood by each participant. Moreover, volunteers were separated by barriers and wore headphones playing white noise. Albeit the Chronos platform can be used to manipulate the on-screen information for each player to implement different interaction topologies, here we focused on a complete topology where all players have access to the current position of all the other group members. The experiment was carried out according to the principles expressed in the Declaration of Helsinki. All participants provided their written informed consent to participate in the study.

**Table 1:** Mean individual frequencies ${\bar{\omega }}_{i}$ in the solo condition.

**Player**	${\bar{\omega }}_{i}$ (**rad/s)**
1	3.40 ± 1.55
2	3.04 ± 0.11
3	6.36 ± 0.58
4	3.34 ± 0.21
5	9.91 ± 0.68
Average	5.21 ± 2.96

*Preprocessing the data*. The position time-series of the players was sampled at 10 Hz, interpolated with a spline to obtain a 100 Hz dataset, and then processed by a Butterworth filter with a cutoff frequency that is twice the typical one associated to human natural movement (~3 Hz). The Hilbert transform (Kralemann et al., 2008) was used to reconstruct the phase associated to each agent from its position time series.

### 4.2. Parameterizing the Models

Comparing Tables 1, 2, we observe that participants reduce the frequency of their oscillations when coordinating with their partners. Independent *t*-tests run between solo and group frequencies in each group showed significant differences [*t*_(11)_ = 17.07, *p* < 0.001]. We observed that the reduction in group frequency was beneficial for coordination, since participants reach a synchronization level significantly different from that obtained when the phases are randomly extracted from a uniform distribution in [0, 2π], that is, *r* = 0.40±0.20.

**Table 2:** Group synchronization frequency ${\bar{\omega }}_{g}$, order parameter $\bar{r}$ and frequency synchronization index ${\bar{\rho }}_{g}$ observed in the experiments.

**${\bar{\omega }}_{g}$ (rad/s)**	** $\bar{r}$ **	** $\bar{\rho }$ **
2.97 ± 0.08	0.90 ± 0.08	0.97 ± 0.01

The standard Kuramoto model (Equation 7) is capable of reproducing the emergence of coordination when agents interact but it fails to capture the observed slowing down in their motions' frequencies. In what follows, we calibrate Models 1–3 and the standard Kuramoto model (Equation 7) on the collected dataset. Then, we perform an ANOVA to assess whether the new models yield a significant improvement over the standard one, and to evaluate which of them is more effective in reproducing the observed reduction in the oscillation frequency among the players when in group.

#### 4.2.1. Tuning the Model Parameters

We follow a different procedure for the standard Kuramoto model and for Models 1–3. As in the standard model the frequencies will always converge to the mean (Bullo, 2018), we select the optimal coupling gain *c*^⋆^ of model (Equation 7) so that the model best captures the observed phase and frequency synchronization level in a mean square sense. Namely, to find the optimal *c*^⋆^, we performed 10 simulations for each candidate value *c*, varied in [0, 2] with step 0.1 with the same duration and sampling as in the experiment. In each simulation, the initial phases were randomly picked from a uniform distribution in [0, 2π], whereas the natural frequency ω_*i*_ of player *i* from a Gaussian distribution with mean and standard deviation corresponding to their sample estimates computed in the solo condition. For the set of simulations, we computed ${\bar{r}}^{\text{sim}}$ and ${\bar{\rho }}^{\text{sim}}$ representing the averages across time and simulated trials of the synchronization metrics *r* and ρ_*g*_ defined in Equations (1) and (4), respectively. Namely, we computed


(12) $$\begin{array}{l} c_{s}^{⋆}=\underset{c}{\arg\;\min}J_{s}(c), \end{array}$$

where


(13) $$\begin{array}{l} J_{s}(c)=\lambda {\left(\frac{{\bar{r}}^{\text{ exp}}-{\bar{r}}^{\text{ sim}}(c)}{{\bar{r}}^{\text{ exp}}}\right)}^{2}+(1-\lambda ){\left(\frac{{\bar{\rho }}^{\text{ exp}}-{\bar{\rho }}^{\text{ sim}}(c)}{{\bar{\rho }}^{\text{ exp}}}\right)}^{2} \end{array}$$

where ${\bar{r}}^{\text{exp}}$ and ${\bar{\rho }}^{\text{exp}}$ are the averages across time and trials of the indexes *r* and ρ. This cost function measures the agreement in phase and frequency synchronization between simulations and experiments. Parameter λ is set to 0.30 to bias parameter choice toward a better agreement on the average ρ, that is, on the level of frequency synchronization.

Models 1–3 have been introduced to also capture the reduced oscillation frequency when in group, therefore a different cost function is needed for tuning their parameters. Let us denote with Π_*m*_, *m*∈{1, 2, 3}, the set of tunable parameters of model *m*. Namely, we have $\Pi_{1}=\left\{c,\tilde{r}\right\}$, Π_2_ = {*c*}, and Π_3_ = {*c*, τ}. Further, we denote as $A_{m}$ the set of admissible values for the parameters of model *m*. Specifically,

- the coupling gain *c* is varied in the range [0, 2] with step 0.1, consistent with the choice made for the standard Kuramoto model;
- the threshold $\tilde{r}$ in Equation (8) is varied in [0.40, 0.95] with step 0.05, where 0.40 is the expected order parameter when the phases of 5 oscillators are randomly extracted in [0, 2π], and 0.95 corresponds to all phases within an angle of π/3 rad;
- the information delay τ is varied in the interval [0.01, 0.35] with step 0.01. The extrema of the interval have been selected on the basis of the transmission delays typically reported in the literature on the sensorimotor system.

**Figure 2:**
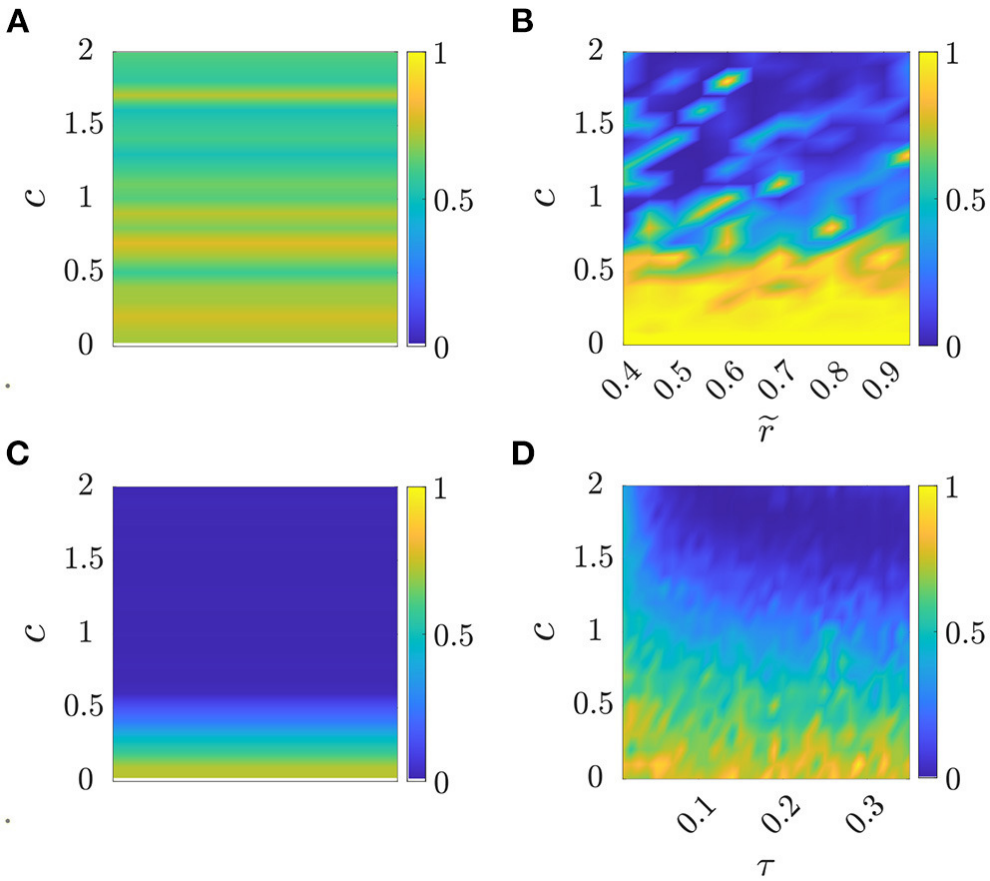
Cost function *J*_*m*_ as a function of parameter selection, see Section 4.2.1. Panel **(A)** corresponds to Standard Kuramoto (Π_*s*_ = {*c*}), **(B)** to Model 1 ($\Pi_{1}=\left\{c,\tilde{r}\right\}$), **(C)** to Model 2 (Π_2_ = {*c*}), and **(D)** to Model 3 (Π_3_ = {*c*, τ}). The values of the cost function *J*_*m*_ are averaged over 10 simulated trials in each parameter set $A_{m}$, see Table 3 for the optimal parameter values for each model.

As in the case of the standard Kuramoto model, we performed ten simulations for each proposed model and combination of parameters. The selection of the initial phases and natural frequencies was performed as above and, for each model, we then computed ${\bar{r}}^{\text{sim}}$ and ${\bar{\rho }}^{\text{sim}}$. In addition, we computed the oscillation frequency ${\bar{\omega }}_{g}^{\text{sim}}$ in the group averaged over time and simulated trials. Then, to calibrate the parameters of model *m*∈{1, 2, 3}, we considered the following cost function:


(14) $$\begin{array}{l} J_{m}(\Pi_{m})=J_{s}(\Pi_{m})+{\left(\frac{{\bar{\omega }}^{\text{ exp}}-{\bar{\omega }}^{\text{ sim}}(\Pi_{m})}{{\bar{\omega }}^{\text{ exp}}}\right)}^{2}, \end{array}$$

where ${\bar{\omega }}_{g}^{\text{exp}}$ is the oscillation frequency in the group averaged over time and experimental trials. Compared with the cost function *J*_*s*_ used to calibrate the standard Kuramoto model, this cost function is complemented by a second term that accounts for the model ability to capture the average group frequency observed in the experiment. The color maps in Figure 2 show for each parameter combination and model the value of *J*_*m*_.

For all models *m*∈{1, 2, 3}, we selected the parameter combination $\Pi_{m}^{⋆}$ yielding the lowest value of *J*_*m*_, that is,


(15) $$\begin{array}{l} \Pi_{m}^{⋆}=\underset{\Pi_{m}\in A_{m}}{\arg\;\min}J_{m}(\Pi_{m}). \end{array}$$

In Table 3, we report the optimal parameter values corresponding to the minimum cost function for each model and dataset.

**Table 3:** Optimal parameter values for each model.

**Standard Kuramoto**	**Model 1**		**Model 2**	**Model 3**	
***c* ^⋆^**	***c* ^⋆^**	** ${\tilde{r}}^{⋆}$ **	***c* ^⋆^**	***c* ^⋆^**	**τ^⋆^**
1.6	1.3	0.55	1.1	1.9	0.17

## 5. Results

**Figure 3:**
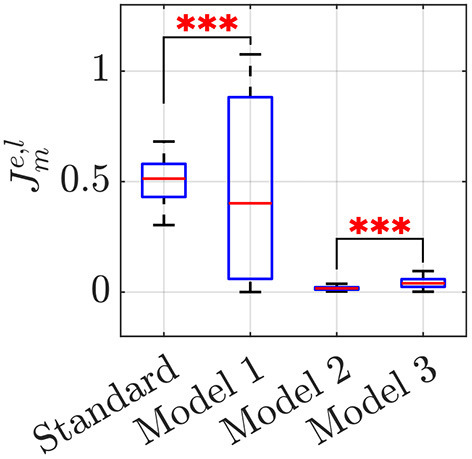
Boxplots of $J_{m}^{e,l}$. Each sample represents the value of the cost function $J_{m}^{e,l}$ for all the possible pairs of experimental and simulated trials, to compare the effectiveness of the *l*-th simulated trial in reproducing the *e*-th experimental data in each model *m*. A triple star corresponds to *p* < 0.001. See Table 4 for the average $J_{m}^{e,l}$ and its standard deviation, in each model *m*.

Here, we compare how each of the models captures the experimental data by evaluating the cost function (Equation 14) for each pair (*e, l*) of experimental and simulated trials. Specifically, denoting *N*_*t*_ the number of trials of the dataset, we compute


(16) $$\begin{array}{l} J_{m}^{e,l}={\left(\frac{\omega_{e}^{\text{ exp}}-\omega_{ml}^{\text{ sim}}(\Pi_{m}^{⋆})}{\omega_{e}^{\text{ exp}}}\right)}^{2}\\ +(1-\lambda ){\left(\frac{\rho_{e}^{\text{ exp}}-\rho_{ml}^{\text{ sim}}(\Pi_{m}^{⋆})}{\rho_{e}^{\text{ exp}}}\right)}^{2}+\lambda {\left(\frac{r_{e}^{\text{ exp}}-r_{ml}^{\text{ sim}}(\Pi_{m}^{⋆})}{r_{e}^{\text{ exp}}}\right)}^{2} \end{array}$$

for all *m*∈{*s*, 1, 2, 3}, *e, l*∈{1, …, *N*_*t*_}, where $\Pi_{m}^{⋆}$ are the optimal parameters reported in Table 3, $\omega_{e}^{\text{exp}}$, $\rho_{e}^{\text{exp}}$ and $r_{e}^{\text{exp}}$ represent group frequency, level of frequency coordination, and order parameter recorded in the *e*-th experimental trial, respectively, whereas $\omega_{ml}^{\text{sim}}$, $\rho_{ml}^{\text{sim}}$ and $r_{ml}^{\text{sim}}$ are the corresponding values in the *l*-th simulated trial. For each model *m*, we evaluated the cost function (Equation 16) for all the 36 possible pairs of experimental and simulated trials, and then ran an ANOVA test on the distribution of $J_{m}^{e,l}$ to compare the effectiveness of each model in reproducing the experimental data. The outcome of the analysis is reported in the boxplot in Figure 3 and Table 4, which show a significant difference among the model performances (*p* < 0.001).

**Table 4:** Results of the ANOVA comparing the cost functions $J_{m}^{e,l}$ for each of the models.

**Anova results**	**Standard kuramoto**	**Model 1**	**Model 2**	**Model 3**
*F*_(3, 134)_ = 60.64, *p* < 0.001	0.51 ± 0.11	0.46 ± 0.37	0.02 ± 0.01	0.04 ± 0.02

*We report the F-statistics, p-value, and the mean and standard deviation of $J_{m}^{e,l}$*.

**Figure 4:**
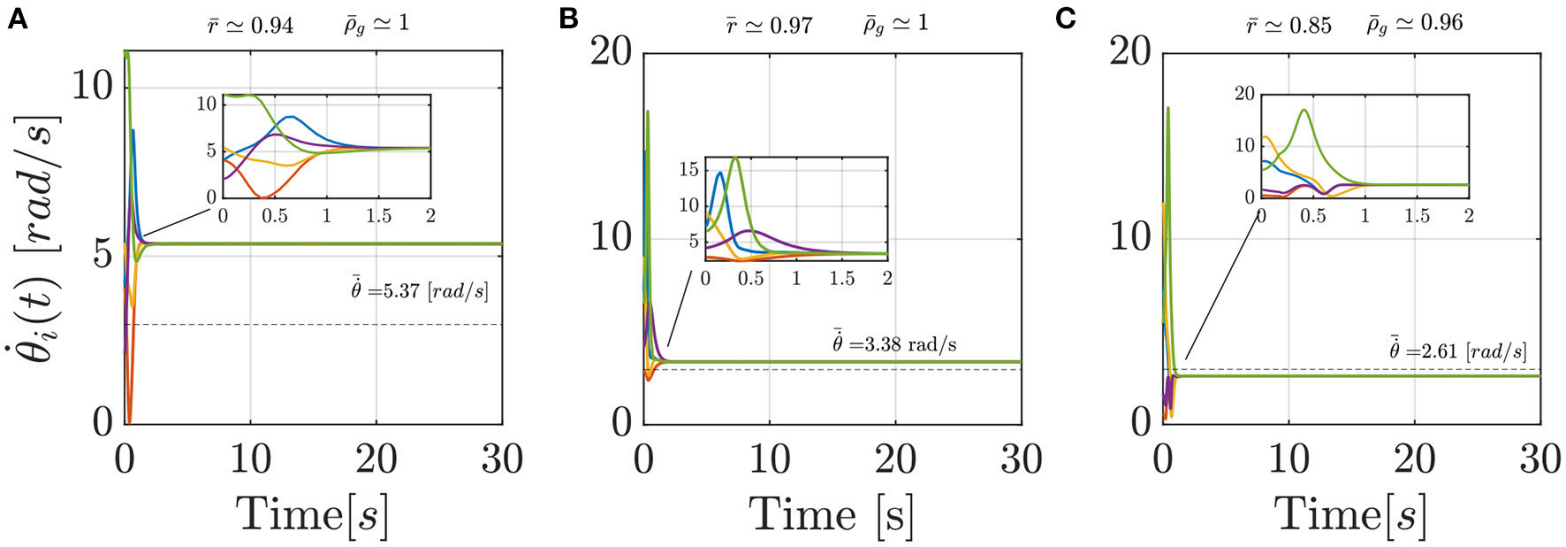
Comparison between three sample simulated trials of the Standard Kuramoto **(A)**, Model 2 **(B)**, and Model 3 **(C)**, respectively. In each panel, the dashed line reports the average experimental group frequency ${\bar{\omega }}_{g}^{\text{exp}}$ whereas on the top the order parameter *r* and the group frequency index ρ_*g*_, in their averaged values, are detailed.

A closer look at the results indicate that Models 2 and 3 should be preferred over the standard Kuramoto, since pairwise comparisons show that they have statistically different performances (*post-hoc* Bonferroni tests, *p* < 0.001), with a notable reduction in the average value of the cost function (Equation 16), see Table 4. Model 1, instead does not prove better than the standard Kuramoto, whereby we cannot reject the null hypothesis of equivalent performances (*p* = 0.50). In terms of average values, Model 2 outperforms also Model 3, albeit their performances are not statistically different. To illustrate how Models 2 and 3 are capable of better matching the average experimental value compared with the standard Kuramoto model, we report in Figure 4 their dynamics in a sample simulated trial.

## 6. Discussion

In this article, starting from the observation that human agents performing a joint oscillatory task together slow down their motion, we proposed three different models to capture this phenomenon based on different biologically-relevant hypotheses underlying sensorimotor group synchronization. The results presented in this manuscript suggest that two models emerge as the ones that better capture the experimental observations, that is, Model 2, which includes a mechanism of selective attention toward the players that are more consistent in their solo conditions, and Model 3, which includes time delays in the dynamics to account for the time needed for information processing. Interestingly, the communication delay estimated from the data by using Model 3 (170 ms) is coherent with the typical delays in the action-perception loop, which includes anticipation, prediction, active preparation and muscular adjustments, in addition to passively added delays in the brain loops. Indeed, the sensorimotor control system requires coordinating different forms of sensory and motor data and these data are generally in various *formats*.

The Kuramoto model has already been successfully used in its simplest form to describe slowing down occuring in human group interaction, e.g., during applause (Néda et al., 2000). Authors of this work were able to explain the key features of applause dynamics but only exploiting parameters tuning. In our work, we propose model extensions to provide possible mechanisms underlying the observed slowing down, which emerges directly through the dynamics. Indeed, differently from the standard Kuramoto model, Models 2 and 3 are capable of capturing not only the observed synchronization level, but also the reduced frequency of oscillation compared to solo trials that we observed in our experiments on a group version of the mirror game. Our findings suggest, therefore, that the observed frequency reduction is due to both selective attention and time delays in the action-perception loop. Thus, both these phenomena should be appropriately taken into account when developing models of group synchronization, as for example in the extended Kuramoto models we propose in this paper. Therefore, we believe that this work represents a valuable contribution for the development of more robust models for the simulation of human group interaction, independent of parameter tuning.

The promising results reported in this manuscript call for further theoretical and experimental research in this area. For instance, from our experiments we could not discriminate which is the dominant effect between the selective attention and the information processing delays. Albeit Model 2 seems to be in average to perform better, we could not reject the hypothesis of equivalent performances with Model 3. Therefore, further experimental studies may be tailored to determine under which circumstances one factor may dominate the other and to assess whether the findings reported in this paper may extend to other kinds of alternative tasks. Finally, albeit our works focuses on steady-state behavior, since in this specific task the agents rapidly converge on their observed oscillation frequency, in other contexts transient dynamics may play a relevant role and should be further investigated.

## Data Availability Statement

The datasets presented in this study can be found in online repository. The name of the repository and accession number can be found below: https://github.com/diBernardoGroup/Modeling-frequency-reduction-in-human-groups-performing-a-joint-oscillatory-task.git.

## Ethics Statement

Ethical review and approval was not required for the study on human participants in accordance with the local legislation and institutional requirements. The patients/participants provided their written informed consent to participate in this study.

## Author Contributions

MB, PD, and BB conceived the study. CC carried out the experiment through the software platform Chronos. CC and PD carried out the modeling and analytical investigations. All authors wrote the paper and reviewed the manuscript.

## Funding

CC was supported by the Italian-French University VINCI Program 2017 that supported her Ph.D. Scholarship. PD was supported by the program STAR 2018 of the University of Naples Federico II and Compagnia di San Paolo, Istituto Banco di Napoli-Fondazione, project ACROSS. BB was supported by grant H2020-FETPROACT-2018-01 EnTimeMent (No 824160). This work was also supported by the Italian Ministry of Research and University through the project ICOSAF under Grant CUP E26C18000180005.

## Conflict of Interest

The authors declare that the research was conducted in the absence of any commercial or financial relationships that could be construed as a potential conflict of interest.

## Publisher's Note

All claims expressed in this article are solely those of the authors and do not necessarily represent those of their affiliated organizations, or those of the publisher, the editors and the reviewers. Any product that may be evaluated in this article, or claim that may be made by its manufacturer, is not guaranteed or endorsed by the publisher.
